# Protocol for a randomised, assessor-blinded, parallel group feasibility trial of flat flexible school shoes for adolescents with patellofemoral pain

**DOI:** 10.1186/s13047-022-00558-z

**Published:** 2022-07-05

**Authors:** Natalie Mazzella, Aaron Fox, Natalie Saunders, Danielle Trowell, Bill Vicenzino, Jason Bonacci

**Affiliations:** 1 Centre for Sports Research, Deakin University, Waurn Ponds, VIC 3215 Australia; 2 Centre for Sports Research, Deakin University, Burwood, VIC 3125 Australia; 3 School of Health and Rehabilitation Sciences, The University of Queensland, St Lucia, QLD 4072 Australia

**Keywords:** Patellofemoral pain, Adolescents, Feasibility, Footwear, Shoes, Knee

## Abstract

**Background:**

There are limited evidence-based treatment options for adolescents with patellofemoral pain (PFP). Flat, flexible footwear have been shown to reduce patellofemoral joint loading and pain in adults with PFP. The efficacy of this intervention in adolescents with PFP is not established. The primary aim of this study is to determine the feasibility of conducting a large-scale randomised controlled trial (RCT) of the effect of flat, flexible school footwear, when compared to traditional school footwear, in adolescents with PFP. The secondary aim is to describe changes in self-reported outcome measures for adolescents with PFP while wearing flat, flexible footwear when compared to traditional school shoes.

**Methods:**

Twenty-four adolescents with PFP will be recruited from the community. Following baseline assessment, participants will be randomly allocated to receive either (i) flat, flexible school footwear or, (ii) traditional school footwear. Participants will wear the shoe as per school requirements throughout a 12-week intervention period. Feasibility will be assessed with (i) ≥ 75% adherence to allocated shoe wear of their total weekly school wear time, (ii) a recruitment rate of one participant per fortnight, and (iii) a dropout rate of ≤ 20%. Patient reported outcome measures will describe changes in knee pain, function, quality of life and global rating of change at 6 and 12 weeks. Descriptive statistics will be used for the primary outcomes of feasibility.

**Discussion:**

This study will determine the feasibility of conducting a large scale RCT evaluating the effect of flat, flexible school shoes for adolescents with PFP. A full-scale study will guide evidence-based management of adolescent PFP.

**Trial registration:**

Australian New Zealand Clinical Trials Registry reference: ACTRN12621001525875, Date registered: 9^th^ November 2021.

**Supplementary Information:**

The online version contains supplementary material available at 10.1186/s13047-022-00558-z.

## Background

There is a significant increase in the number of musculoskeletal injuries reported at the onset of and during adolescence [1]. A quarter of adolescents aged 12–15 years’ experience knee pain, with patellofemoral pain (PFP) the most prevalent diagnosis [2]. PFP is characterised by pain in and around the patella that is aggravated by weightbearing activities such as running and stair ambulation [3]. PFP in adolescence has substantial implications on long-term physical and mental health [4]. Seventy-percent of adolescents with PFP are likely to cease or reduce their participation in physical activity, compared to 50% of adolescents with other types of knee pain [5]. Adolescents with PFP experience poorer mental health and they are twice as likely to use pain medication regularly when compared to other diagnoses of knee pain [6, 7]. The long-term prognosis of adolescent PFP is poor, with symptoms persisting in up to 91% of adolescent cases after 4–18 years [8, 9].

Despite the burden of adolescent PFP, there is a paucity of studies that have examined treatment options within this cohort. Treatment of adolescent PFP is generally adopted from guidelines implemented in adult studies [10]. As reported in the ‘Best Practice Guide to Conservative Management of PFP’ and an international consensus statement, exercise therapy has been shown to improve pain in the short, medium, and long-term in adults with PFP [10, 11]. However, the efficacy of exercise therapy for adolescents with PFP is not as effective as that reported in adult studies [12]. Matched studies examining exercise therapy in adults and adolescents with PFP reflect this, with 62% of adults reportedly recovering compared with only 38% of adolescents [13, 14]. Several factors may underpin this. Exercise therapy is designed to address strength deficits that are observed in adults with PFP, such as reduced hip abduction and external rotation strength [15, 16]. Adolescents with PFP do not demonstrate hip and knee strength deficits when compared to healthy controls [17, 18]. Therefore, exercise therapy targeted to address strength deficits may be less efficacious in adolescents.

Poor adherence to exercise-based interventions are also reported in adolescents with PFP due to factors such as school commitments, time constraints and boredom with the program [12, 19, 20]. Adolescents with PFP also typically report pain in both knees, with up to 79% reporting bilateral symptoms of PFP compared to 43% of adults with PFP [21]. This bilateral nature may increase the time commitment of prescribed exercise therapy [12, 21]. Given adolescents with PFP do not demonstrate reduced muscular capacity, and their adherence to prescribed exercise therapy may be reduced, alternative treatment options that focus on patellofemoral joint load reduction may be more advantageous within this population.

Flat, flexible footwear have been shown to reduce patellofemoral joint loading during running [22–24], stair descent [25] and jumping [26] in asymptomatic adults as well as adults with PFP [27]. Improvements in PFP symptoms among adults have also been reported when flat, flexible footwear have been used in isolation [27] or when combined with gait retraining [28]. The effectiveness of flat, flexible footwear on patellofemoral joint loads, pain, and function in an adolescent PFP cohort is unknown. Adolescents spend a large proportion of their weekdays attending school in school footwear [29]. School footwear must adhere to school uniform guidelines that most commonly include a low-heeled, leather upper, lace-up or buckle shoe [30]. Activity data shows that adolescents participate in a range of sport and physical activity while at school and 23% meet their daily moderate-vigorous activity targets within school breaks alone [31]. This suggests that a high percentage of total daily physical activity is performed in school footwear during school hours [32, 33]. Wearing a flat, flexible shoe at school may be an alternative management option for adolescents with PFP as shoes address the bilateral nature of adolescent PFP; are likely to have greater adherence than exercise therapy; and are usually worn by adolescents for long periods of time while at school.

Investigating the feasibility of a flat, flexible school shoe for adolescents with PFP is a necessary step toward developing targeted evidence-based management. Early intervention is essential for minimising the potential for chronicity within this population, as well as reducing the significant long-term health-related behaviour changes associated with adolescent PFP [5, 9]. A feasibility trial provides the first step to determining the requirements and potential application of a future large scale randomised controlled trial (RCT) [34]. The primary objective of this study is to determine the feasibility of conducting a large scale RCT on the effect of a flat, flexible school shoe in adolescents with PFP. The secondary outcome is to describe changes in knee function and pain with the use of a flat, flexible school shoe compared to a traditional school shoe in adolescents with PFP.

## Methods

### Experimental/trial design

This study is an assessor blind, randomised, feasibility trial, with two parallel groups of adolescents with PFP. The research proposal has been developed in consultation with the Standard Protocol Items: Recommendations for Interventional Trials (SPIRIT) 2013 statement [35] and the Consolidated Standard of Reporting Trials (CONSORT) 2010 guidelines for randomised pilot and feasibility trials [36].

Ethical approval was granted through the Deakin University Human Research Ethics Committee (2021–135). The trial was prospectively registered on the Australian and New Zealand Clinical Trials Registry (ACTRN12621001525875, Date registered: 9^th^ November 2021). Written informed consent will be obtained from all participants and their parents/guardians prior to participation within the study.

### Participants

Male and female adolescents will be eligible for inclusion if they meet the following criteria: (i) aged between 12–18 years, (ii) have PFP from a non-traumatic onset of at least six weeks duration, (iii) have pain ≥ 30/100 mm (mm) on a visual analogue scale (VAS), and (iv) have knee pain which is aggravated by activities that load the patellofemoral joint (e.g., squatting, stair ascent or descent, running).

Adolescents will be excluded if they (i) have pain at sites other than the anterior knee (e.g., other knee structures, hip, pelvis, lumbar spine), (ii) have a history of hip, knee or spine surgery, or other suspected knee joint pathology (e.g., Sinding Larsen Syndrome, Osgood Schlatter’s Disease), (iii) have planned lower limb surgery, (iv) have a neurological condition or systemic arthritis, (v) are currently wearing flat flexible footwear for school and/or (vi) have any condition which prevents them from wearing flat flexible footwear (e.g., calcaneal apophysitis).

Adolescent volunteers will be recruited from the community using a targeted comprehensive recruitment strategy with proven efficacy in previous studies of PFP [28, 37, 38]. Recruitment strategies will include paid social media advertising, dissemination through social media networks and flyers displayed at local sports medicine and allied healthcare clinics, footwear stores and sporting clubs/recreational facilities where PFP is likely to be prevalent. Eligibility criteria are based on previous high quality RCTs for PFP [13, 37].

To observe feasibility outcome of adherence, we plan to recruit 24 participants with PFP. A minimum of 23 participants are required to observe the feasibility outcome of adherence ≥ 75% allocated shoe wear indicating progression to a full trial is feasible. If adherence to allocated shoe wear is ≤ 50% (i.e., two school days per week, excluding a sporting day) progression to a main trial is not feasible (alpha < 0.05, β 0.2) [37, 39, 40].

### Study procedures

Following contact with the research team, potential participants will be telephone screened for inclusion and exclusion criteria. Participants will then undergo a physical assessment at Deakin University to confirm diagnosis of PFP and the exclusion of other diagnoses of knee pain (e.g., Osgood Schlatter’s disease). All screening procedures will be performed by a registered podiatrist (NM) with more than nine years of clinical practice experience who has undergone additional training in PFP diagnosis by an experienced physiotherapist (JB).

Eligible participants will provide written consent to participate along with their parent or guardian for those under 18 years of age. Following baseline data collection eligible participants will be randomised to receive either (i) a flat, flexible school shoe, or (ii) a traditional school shoe to be worn throughout the 12-week study period. Randomisation procedures will be performed by a researcher external to this project.

**Fig. 1:**
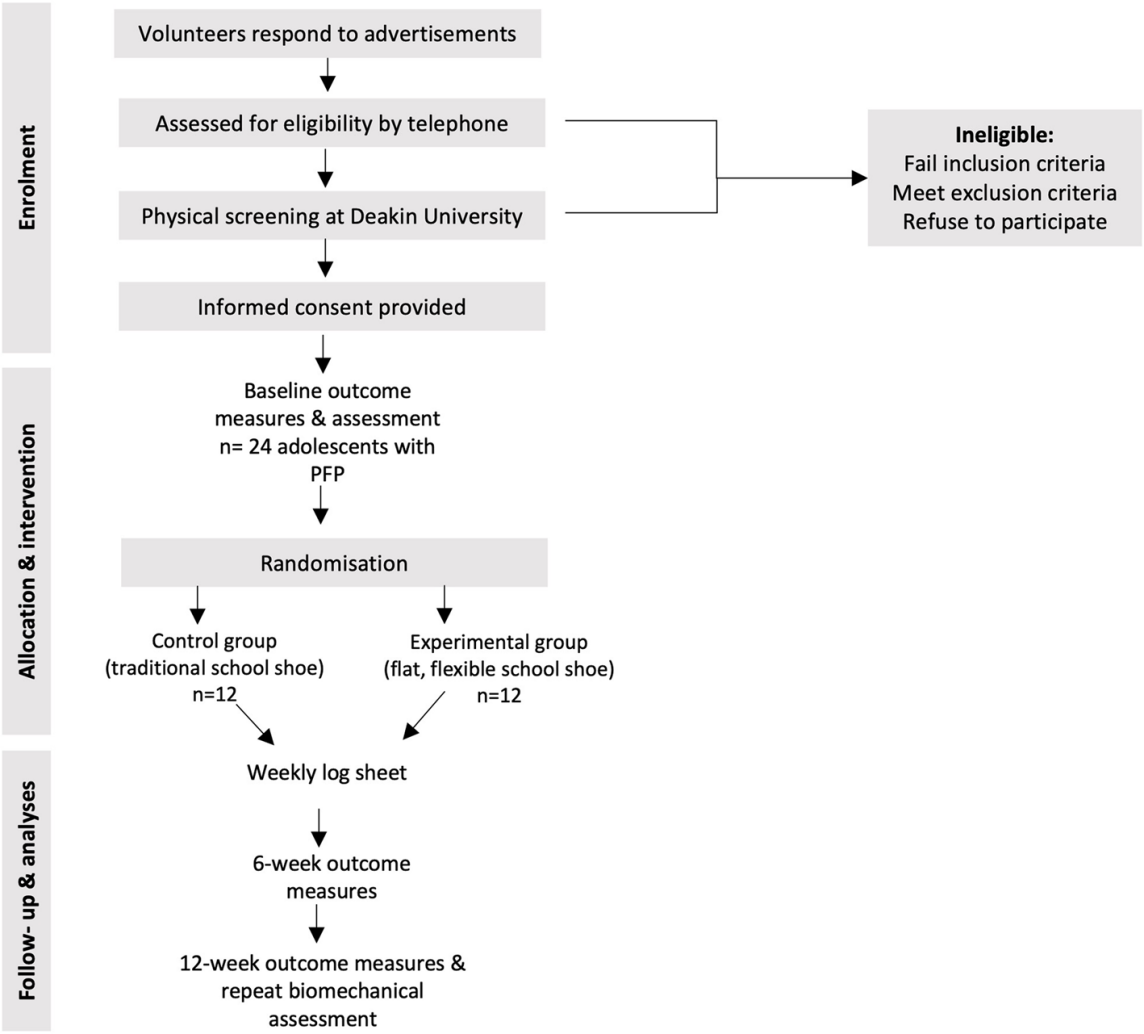
Participant flow through the study

As the outcomes within this study are patient reported, this study is considered assessor-blinded which is consistent with other RCTs utilising footwear interventions [39]. The research team processing the participant-reported data will be blinded to group allocation. Due to the inability to blind participants to the shoe they are wearing, participants will not be informed of the hypotheses of the study, nor of the differences between the shoes [39]. They will simply be recommended to wear the shoe at all times they would usually wear their school shoe. Participants will be able to keep their allocated shoe at the cessation of the trial. Participant flow through the study is outlined in Fig. 1.

### Randomisation

Randomisation procedures will be performed via fixed concealed allocation. The randomisation sequence will be computer generated with permuted blocks of four or six participants. All assessors responsible for measuring and analysing key outcomes will be blinded to participant allocation.

### Interventions

Participants will be fitted into both types of footwear at baseline by a podiatrist (NM) with nine years of clinical and footwear fitting experience to ensure they are comfortable and correctly fitted [41]. At this time, participants will be issued with an information sheet (see Additional file 1) outlining important information about their shoes, the study, and the requirements of participation within the study. Participants will be advised to wear their allocated shoe for the duration of time per week they would normally wear their school footwear.

#### Flat, flexible school shoe

**Fig. 2:**
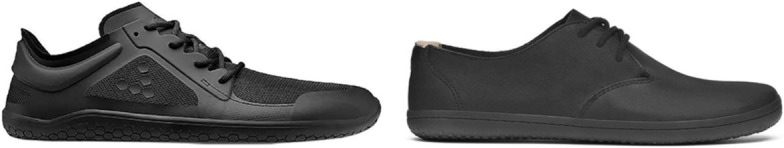
Flat, flexible footwear to be used within study, Vivobarefoot Primus Lite (left) & Vivobarefoot RA II (right) (Vivobarefoot, Freiburg, Germany)

Participants randomised to flat, flexible school shoes will receive the Vivobarefoot Primus Lite and/or the Vivobarefoot RA II (Fig. 2), which are both commercially available flat, flexible black lace up shoes (Vivobarefoot, Freiburg, Germany). The Primus Lite and the RA II are lightweight, have a 3 mm outsole, zero heel-toe offset, a mass of 180 g, and no stability or motion control features. The Primus Lite and the RA II score 25/25 on the minimalist shoe index [42]. Participants will receive either the RA II or the Primus Lite dependant on shoe size and stock availability. Participants will wear this shoe as per school requirements throughout the 12-week intervention period.

#### Traditional school shoe

**Fig. 3:**
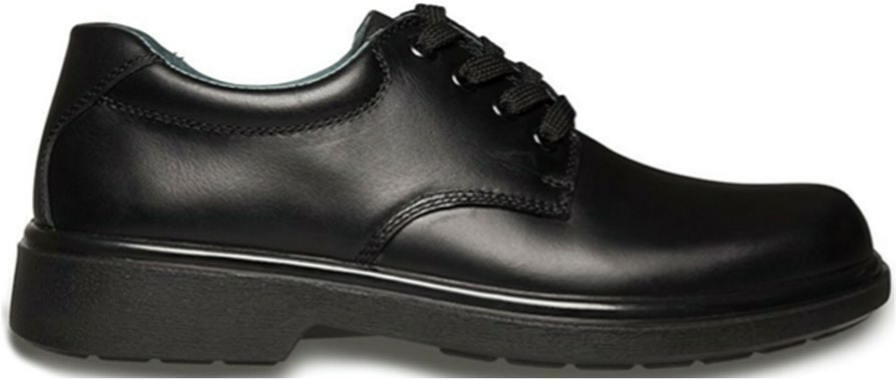
Traditional school shoe to be used within study, Clarks Daytona (Clarks, Street, England)

Participants randomised to the traditional school shoe will receive a pair of Clarks Daytona (Fig. 3) (Clarks, Street, England). The Clarks Daytona has a stiff midsole and heel counter, a 12 mm heel-toe offset, and mass of 350 g. The Clarks Daytona scores 2/25 on the minimalist shoe index indicating a low degree of minimalism and flexibility [42].

### Concomitant care

If participants are taking medication for their knee pain, they will be permitted to continue this throughout the study duration. This is consistent with other studies performed in adolescents with PFP [13, 37]. At the time of entry within the study, participants will be asked to refrain from commencing new treatments for their knee pain for the duration of the study and to avoid using other assistive devices such as braces or orthotics for the study duration. Participants will be asked to report any use of co-interventions in the weekly log sheet (see Additional file 2).

### Outcome assessment

Once consent is provided, baseline testing will follow at the Deakin University 3D Gait Laboratory. The duration of testing will be approximately 1.5 h. Baseline information will be obtained from participants including demographic data, body mass and height, sex, affected knee/s, duration of symptoms, previous treatments, and aggravating activities [43]. To assess the stage of adolescence, participants will complete the modified Pubertal Maturational Observational Scale at baseline [44]. This scale has been used in other studies involving adolescents and can be used to reliably classify adolescent developmental stages [45–47].

Participants will complete self-reported outcome measures at baseline, six weeks, and 12 weeks. Data collection will be performed through self-reported questionnaires performed via Qualtrics™ (Qualtrics, Provo, United States of America). Throughout the duration of the study participants will be asked to keep a weekly log (see Additional file 2) of the type of shoe worn that day; hours spent wearing that shoe; any adverse events associated with the allocated school shoe; use of co-interventions (e.g., pain medication, other footwear, taping); and any other comments. Participants will complete this in an online format distributed to them weekly via Qualtrics™. If participants do not have the equipment required to access the online format, a hard copy will be provided.

### Outcomes measures

#### Primary outcome measures

The primary outcome from this study is to determine the feasibility of conducting a full-scale RCT in adolescents with PFP. Feasibility will be assessed by the following outcome parameters; (i) adherence to allocated shoe wear of ≥ 75% of their total weekly school shoe wear time, (ii) a recruitment rate of one participant per fortnight, and (iii) a dropout rate of ≤ 20%. Success of blinding and participants’ expectations of treatment will be evaluated using the Credibility and Expectancy Questionnaire [48]. This will be completed at the end of the baseline assessment immediately after participants have been fitted into their shoes and then at the end of week one [37].

#### Secondary outcome measures

Secondary outcome measures will include the following patient reported outcomes taken at baseline, six weeks, and 12 weeks.

*Knee Pain Severity* will be assessed using a 100 mm VAS, with 0 mm indicating no pain and 100 mm indicating the worst pain imaginable. Participants will be asked to report their worst pain and usual pain in the past week. The VAS for usual or worst pain has been shown to be reliable and valid in assessing treatment outcomes in PFP [49].

*Knee Injury and Osteoarthritis Outcome Score- Child Version (KOOS-Child)—*The KOOS-Child is a patient reported outcome measure assessing (i) pain; (ii) symptoms; (iii) difficulty during daily activity; (iv) function in sports and play; and (v) knee-related quality of life [50]. Participants respond to each item using a 5-point Likert scale from 0 (no problem) to 4 (extreme problems). The scores are combined and displayed on a 0–100 scale with 0 indicating no problem and 100 indicating extreme knee problems. The KOOS-Child is recommended to evaluate knee function in adolescents and young people with a broad range of knee pain [51].

*Knee Injury and Osteoarthritis Outcome Score- Patellofemoral Pain (KOOS-PF)—*The KOOS-PF is a patient reported subscale of the KOOS for use in patients with PFP and patellofemoral osteoarthritis. This subscale of the KOOS is designed to be used in conjunction with the KOOS and/or KOOS-Child and has 11 items with the same response scales. The KOOS-PF has been shown to be valid and reliable when tested in adults but has yet to be assessed in adolescents and young people [52]. The KOOS-PF has been used by other RCTs conducted on adolescents, therefore it has been selected to ensure consistency of outcome measure assessments [37].

*Anterior Knee Pain Scale (AKPS)—*The AKPS is a patient reported assessment of 13 items related to symptoms and functional limitations. The AKPS is scored from 0 to 100 with lower scores indicating greater pain and functional limitations. The AKPS has been shown to be reliable and valid in assessing treatment outcomes in PFP [49].

*Youth Quality of Life Short Form (YQOL-SF)—*The YQOL-SF is a reliable tool used to assess the generic quality of life in adolescents aged 11–18 years with and without chronic conditions or disability [53]. The short form, derived from the Youth Quality of Life- Research, measures four domains including sense of self, social relationships, environment, and general quality of life. Participants respond to several statements on a scale from 0 (not at all) to 10 (completely). The total participant score is then transformed with a higher score indicating a better self-reported quality of life.

*Global Rating of Change (GROC)—*A 7-point Likert scale will be used to evaluate GROC at six weeks and 12 weeks [54]. Participants will be asked how their knee pain has changed since the start of the trial using the following responses: ‘completely recovered’, ‘strongly recovered’, ‘slightly recovered’, ‘same’, ‘slightly worse’, ‘much worse’, and ‘worse than ever’. The GROC has been used as an outcome measure in previous RCTs of adolescents with PFP [13, 37].

*Biomechanical analysis—*Lower limb kinematics and kinetics will be measured while walking and running on an instrumented treadmill (Bertec, Ohio, United States of America) at baseline and 12 weeks. Participants walking and running biomechanics will be assessed while wearing the traditional school shoe; flat, flexible school shoe; and a standard athletic shoe (Asics Gel Cumulus 16 [Asics, Kobe, Japan]) at baseline and their allocated school shoe and the standard athletic shoe at 12 weeks. Outcome measures will include: (i) hip, knee and ankle joint angles and torques in the sagittal, frontal, and transverse planes and (ii) patellofemoral joint forces. Biomechanical analysis will be used to understand the immediate (*within session)* and short-term (*12 weeks)* effects of flat, flexible school footwear on lower limb kinematics and kinetics and patellofemoral joint loads.

### Adverse events

For the study duration participants will be advised to report information on adverse events and/or use of concomitant care within their weekly log sheet. Participants will be encouraged to report any discomfort they experience to the research team. If required, participants will attend an additional appointment with the research team to discuss any discomfort they may be experiencing. In this instance, standard clinical practice principles will be applied. The researcher may recommend strategies to improve the adaptation (e.g. a return to their regular footwear until pain settles). These events will be recorded as adverse events and if the discomfort cannot be reduced or tolerated, the participant will be encouraged to return to using their normal footwear and the individual’s participation with the intervention will be ceased.

### Use of co-interventions

Participants will be asked to report any use of co-interventions within their logbook (e.g., pain medication, taping). This will be recorded in their logbooks over the three-month period. The reporting of co-interventions is common in other trials of adolescent PFP [37].

### Planned statistical analysis

Data processing, data entry and data analysis will be performed by an assessor who is blinded to group allocation. All statistical analysis will be performed using SPSS version 27.0 or later (SPSS, Chicago, USA). Descriptive statistics will be used for the primary outcomes of feasibility and reported in relation to the pre-specified feasibility criteria. Patient reported outcome measures will be described with means and standard deviations for continuous data and counts and percentage for categorical data.

### Data management

Data gathered throughout the study will be coded in a re-identifiable format and stored on a separate database to group identifier to maintain blinding of the primary investigator. All electronic data will be stored on a shared drive of password protected computers.

## Discussion

PFP has substantial implications on long term health and physical activity behaviours in adolescents [3, 8]. Adolescent knee pain is associated with significant health-related consequences, with PFP believed to carry the worst prognosis when compared to other diagnoses of adolescent knee pain (e.g., Osgood Schlatter’s Disease, Sinding Larsen Syndrome) [5, 6, 9]. Adolescents with PFP are likely to reduce or cease participation in recreational activity and report chronic pain that persists into adulthood, when compared to other diagnoses of adolescent knee pain [6]. Current treatment for PFP is designed to address features of PFP that are seen in adults, such as reduced hip and knee strength [15, 16]. However, these strength deficits are not seen in adolescents with PFP, and recommended treatment has shown less efficacy when compared to adults [13, 14].

Early intervention of adolescent PFP may provide a solution to addressing the poor long-term prognosis of this condition (9). Footwear provides an opportunity to explore a treatment option that may better suit an adolescent cohort. Shoes are a requisite for most school uniforms and adolescents spend a large percentage of their weekday time at school [29]. Activity data shows that adolescents participate in a range of sport and physical activity while at school [31]. A school shoe intervention for adolescents with PFP may help to address adherence issues commonly seen with exercise therapy [19, 20].

Flat, flexible shoes may be an appropriate alternative for school use, but the clinical effects of this footwear in an adolescent cohort are unknown. Studies in adults with PFP and medial tibiofemoral osteoarthritis demonstrate this style of footwear is safe to use and have little adverse effects [28, 39, 55]. Within a RCT of 56 older women with medial tibiofemoral osteoarthritis, flat, flexible footwear were effective at reducing pain, improving function on activities of daily living, and reducing daily analgesic intake when compared to traditional footwear [55]. Similarly, a 36% reduction in pain on the Western Ontario and McMaster Universities Osteoarthritis Index scale was seen in 16 adults with medial tibiofemoral osteoarthritis when using flat, flexible footwear compared to traditional stiff soled footwear [56]. In contrast, a larger scale RCT of 164 adults with medial tibiofemoral osteoarthritis reported improved knee pain during walking in stable supportive shoes compared to flat, flexible shoes [39]. It is not appropriate to generalise findings from adults to adolescents with PFP and studies are needed in the target population to develop evidence-based clinical guidelines [12]. Flat flexible shoes are widely available, cost effective, easy to use and there is a minimal risk of adverse events associated with their use [55]. Studies show that they have good compliance to daily wear of at least six hours over six months in adults [39]. As footwear is regularly worn by adolescents to school, a footwear intervention may allow adolescents with PFP to self-manage their pain during activities of daily living and physical activity while at school.

The primary objective of this study is to determine the feasibility of conducting a large scale RCT on the effect of a flat, flexible school shoe in adolescents with PFP. Large scale RCTs are needed to allow the development of evidence-based guidelines specific to an adolescent PFP cohort. This study has been designed in consultation with the SPIRIT and CONSORT statements for randomised trials [35, 36]. Strengths of this study include the planned randomisation of participants, blinding of outcome assessors and a clear range of measurable feasibility outcomes [34, 40, 57]. Investigating the feasibility of a flat, flexible school shoe for adolescents with PFP is a necessary step toward developing early and targeted evidence-based management. Secondary outcome measures have been selected based on their use in other studies of adolescents with PFP as well as their clinical applicability and reproducibility within an adolescent cohort [13, 37].

## Supplementary Information

**Additional file 1.**

**Additional file 2.**

## Data Availability

De-identified individual participant data will be collected during the trial. Access to the data will be subject to approvals by the Principal Investigator with a requirement to sign a data access agreement.

## Abbreviations

AKPS: Anterior Knee Pain Scale
CONSORT: Consolidated Standards of Reporting Trials
GROC: Global Rating of Change
KOOS: Knee Injury and Osteoarthritis Outcome Score
KOOS-PF: Knee Injury and Osteoarthritis Outcome Score – Patellofemoral Subscale
PFP: Patellofemoral pain
RCT: Randomised controlled trial
SPIRIT: Standard Protocol Items: Recommendations for Interventional Trials
VAS: Visual analogue scale
YQol-SF: Youth Quality of Life- Short Form
