# Supplementary material for: Protocol for a randomised, assessor-blinded, parallel group feasibility trial of flat flexible school shoes for adolescents with patellofemoral pain
Source: J Foot Ankle Res. 2022 Jul 5;15:52. doi: 10.1186/s13047-022-00558-z (PMC9254449; doi:10.1186/s13047-022-00558-z)
Supplement: Supplementary file 1 — Additional file 1. [file 13047_2022_558_MOESM1_ESM.pdf]

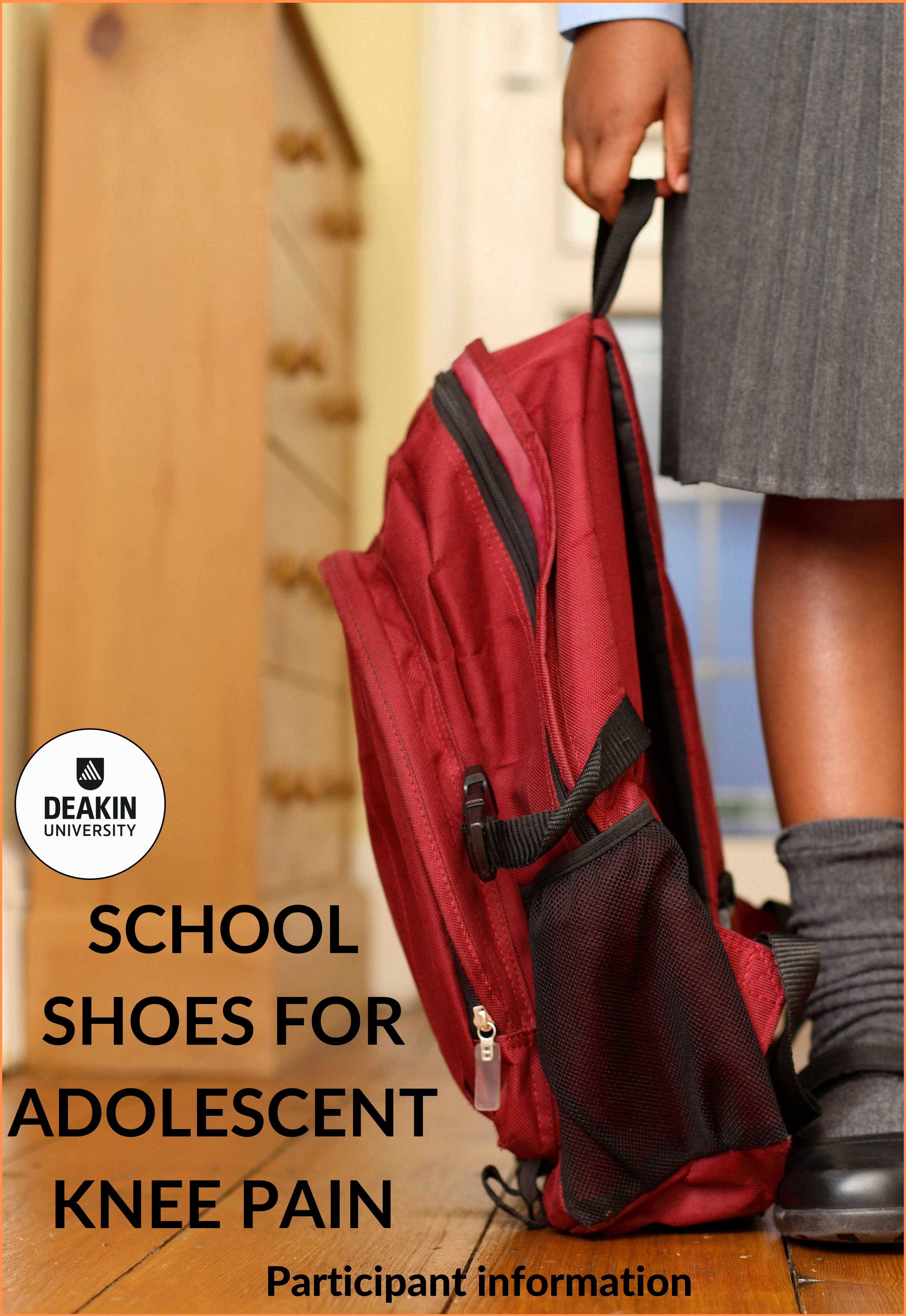

DEAKIN UNIVERSITY

# SCHOOL SHOES FOR ADOLESCENT KNEE PAIN

**Participant information**

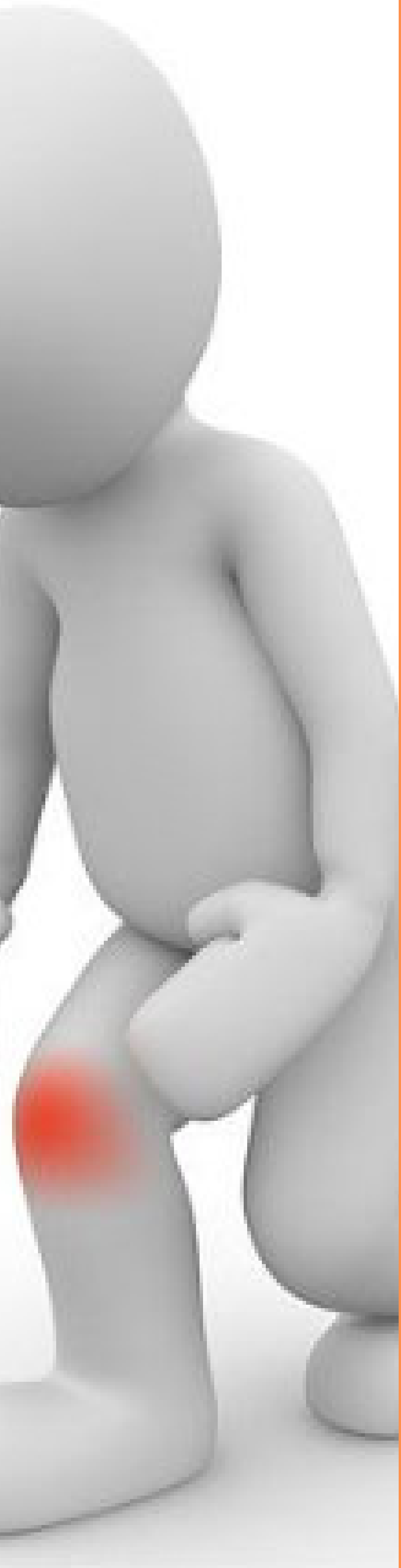

# WHY IS KNEE PAIN SUCH A PROBLEM FOR SECONDARY SCHOOL STUDENTS?

Have you ever noticed how much of a bother having a sore knee can be? Knee cap pain, or pain at the front part of your knee, can be aggravated by activities such as running and jumping, but it can also get sore after doing nothing- even sitting down can sometimes make it sore.

Did you know that as many as one in four adolescents can experience pain in their knee? We know that having a sore knee can stop you from taking part in P.E and playing sport or participating in recreational activities. Pain at the front of your knee can also last for a very long time. Therefore, finding treatment that helps reduce the pain and get you back to being active is vital.

Some studies suggest that different types of shoes can help adults with kneecap pain. We are interested to know if different types of school shoes can help your knee pain. Since you are required to wear school shoes to school, we believe this is a great treatment option for your knee pain.

The only thing you need to do with this treatment is remember to wear them on the days you would usually wear your school shoes!

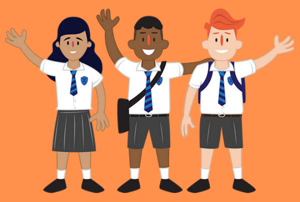

# WHAT HAPPENS WHILE I AM IN THE STUDY?

During your first visit to Deakin University, we will assess you walking and running in different shoes and ask you to complete a questionnaire about your knee pain. This will take around 90minutes and at the end of the session you will be fitted by a Podiatrist into a pair of new school shoes to take home and wear over the next 12 weeks.

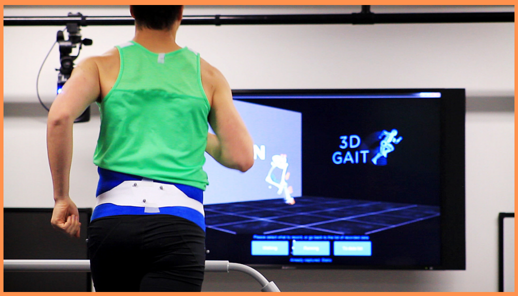


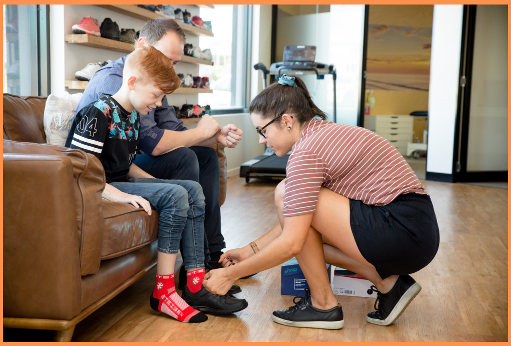

At the end of the first visit, you will be given a pair of black school shoes to take home and to wear to school. These shoes might feel a bit different to what you may usually wear for school. Your shoes should fit and feel perfectly. If they don't, then please let Natalie know so we can help you feel comfortable. We don't want your feet to be unhappy. You should wear these on the days you would normally wear your shoes to school for the entire 12 week period. It is really important that you wear them over the 12 weeks so that you are able to get the most benefit from them and so that we can understand how they are helping with your knee pain.

**1st appointment (Week 1**

- Running assessment
- Receive shoes
- Fill out questionnaire

**Half way (6 weeks)**

- Answer same questionnaire from Week 1

**End of study (12 weeks)**

- Running assessment at Deakin
- Answer same questionnaire from Week 1

| | Monday | Tuesday | Wednesday | Thursday |
|---|---|---|---|---|
| What school shoes did you wear today? | | | | |
| How many hours did you spend wearing your school shoes today? | | | | |
| Did you have any problems in your legs or feet while wearing your school shoes today? | | | | |
| What activities or sport did you perform today? | | | | |
| Did you take any medication (e.g. Panadol, Nurofen) or use anything for your knee pain today (e.g. braces, taping, shoe inserts)? | | | | |

While you are in the study, you will be required to fill out a weekly log sheet (like the one on the left) of when you wore the school shoes, as well as a set of questions halfway through the study and again at the end. These questions will be the same as the questions you filled out during the first visit. It is really important that you fill out the log sheet each week so that we can understand how the shoes benefit you the most. If you feel pain in your foot or other areas in your legs, please contact Natalie.

# A FEW EXTRA THINGS

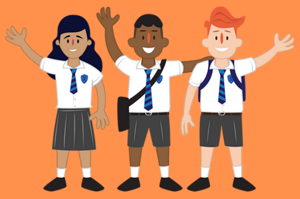

## CAN I HAVE OTHER TREATMENT WHILE I AM IN THE STUDY?

While you are in the study, we would like you to avoid starting any new treatments for your knee pain. This might include orthotics, braces or injections. We need you to do this so we can best understand if the shoes are helpful for adolescents with knee pain. If you take any medication for your knee pain, it is also important you include this in your weekly log sheet.

## WHAT WILL THE SHOES FEEL LIKE?

The shoes you receive may feel a little bit unfamiliar when you first receive them- much the same as when you get a new pair of school shoes. They should fit and feel perfectly and you shouldn't experience any slipping in the heel or any squishing of your toes. If this is happening, please contact Natalie.

## CAN I PLAY SPORT AT SCHOOL IN THESE SHOES?

We want you to use the shoes as you would normally use your school footwear. If you normally wear runners for sport or on the days you have PE you should continue to do so. You are welcome to play sport in your allocated school shoe at lunch time if this is what you usually do with your school shoes.

## WHAT DO I HAVE TO DO AS A PARTICIPANT IN THIS STUDY?

- Wear the shoes as often as you would wear your school shoes
- Fill out the weekly log sheet
- Report any discomfort or concerns you have to Natalie
- Fill out a questionnaire half way through the study (and again at the end)
- Come in for one session of testing at the end of the 12 weeks
- Keep the shoes you have been given at the end

## DO I WEAR THE SHOES ON SCHOOL HOLIDAYS?

We don't expect you to wear the shoes on school holidays. However, if they are helping with your knee pain you are welcome to continue wearing them- just be sure to make note of this in your weekly log sheet. Once you return from school holidays, you should start wearing them as normal again.

## WHO DO I CONTACT IF I HAVE ANY PROBLEMS?

Natalie Mazzella - nmazzella@deakin.edu.au
