# Supplementary material for: Protocol for a randomised, assessor-blinded, parallel group feasibility trial of flat flexible school shoes for adolescents with patellofemoral pain
Source: J Foot Ankle Res. 2022 Jul 5;15:52. doi: 10.1186/s13047-022-00558-z (PMC9254449; doi:10.1186/s13047-022-00558-z)
Supplement: Supplementary file 2 — Additional file 2. [file 13047_2022_558_MOESM2_ESM.pdf]

Weekly log sheet

Weekly log sheet
We would like you to complete this diary at the end of each week. There are no right or wrong answers to the questions- we simply want to know what shoes you wore and how you felt. You are welcome to fill this out with the help of your parent or guardian.

Please write your participant ID (from the email sent to you)

____

Please write Monday's date:

____

Please tick which days you were meant to wear your school shoes, and on those days please write which shoe you wore.

- ☐ Monday ________________________________
- ☐ Tuesday ________________________________
- ☐ Wednesday ________________________________
- ☐ Thursday ________________________________
- ☐ Friday ________________________________

For the days when you did not wear your school shoes, please write which shoe you wore to school (such as runners or casual footwear).

- ☐ Monday ______________________________
- ☐ Tuesday ______________________________
- ☐ Wednesday ______________________________
- ☐ Thursday ______________________________
- ☐ Friday ______________________________

Please write how many hours you spent wearing your school shoes on each day this week.

- ☐ Monday ______________________________
- ☐ Tuesday ______________________________
- ☐ Wednesday ______________________________
- ☐ Thursday ______________________________
- ☐ Friday ______________________________

Please list any problems you experienced in your legs or feet while wearing your school shoes on each day this week.

- ☐ Monday ______________________________
- ☐ Tuesday ______________________________
- ☐ Wednesday ______________________________
- ☐ Thursday ______________________________
- ☐ Friday ______________________________

Please list what activities or sport you performed on each day this week.

- ○ Monday ______________________________
- ○ Tuesday ______________________________
- ○ Wednesday ______________________________
- ○ Thursday ______________________________
- ○ Friday ______________________________
- ○ Saturday ______________________________
- ○ Sunday ______________________________

Please list any medication (e.g. Panadol, Nurofen) or anything you used for your knee pain (e.g. braces, taping) on each day this week.

- ◯ Monday ____
- ◯ Tuesday ____
- ◯ Wednesday ____
- ◯ Thursday ____
- ◯ Friday ____
- ◯ Saturday ____
- ◯ Sunday ____

Please list all shoes you wore over the weekend in the text box below:

____

Use this space to add in any additional information you would like the research team to know from this week.

____
